# Neuroprotection exerted by ischemic preconditioning in rat hippocampus involves extracellular signal receptor changes

**DOI:** 10.1186/2193-1801-4-S1-P21

**Published:** 2015-06-12

**Authors:** Jan Lehotsky, Maria Kovalska, Barbara Tothova, Zuzana Tatarkova, Peter Kaplan

**Affiliations:** Comenius University, Jessenius Faculty of Medicine in Martin, Slovakia

**Keywords:** ischemic/reperfusion injury, preconditioning, ERK, rat

Ichemic-reperfusion injury induced by four vessel occlusion affects vulnerable hippocampal CA1 (cornus ammonis 1) pyramidal neurons. Ischemic tolerance evoked by preconditioning (IPC) represents a phenomenon of CNS adaptation to any subsequent ischemia. We refer here for the changes in the external signal receptor protein kinase pathways of the hippocampal area following by IPC. Ischemia was induced by a 4-vessels occlusion (4VO) and the rats were preconditioned by a non-injurious ischemia. Apoptotic markers were used to follow the degeneration process. Western blot and immunohistochemistry identified phosphorylated extracellular signal-regulated protein kinase and p38 proteins in injured hippocampal areas. P-ERK quantification increased after IPC and reached the highest level at 24 h after ischemia. Interestingly, neuroprotection induced by IPC lead to the opposite effect on MAPK/p38, where the level was lowest at 24 h after ischemia. The study clearly shows that phosphorylated form takes part in complex cascades triggered by IPC in the selectively vulnerable hippocampal region. In addition, study reveals an interplay between p-ERK and p-p38 which participates in the tolerence mechanisminduced by preconditioning.

